# Snacks contribute considerably to total dietary intakes among adults stratified by glycemia in the United States

**DOI:** 10.1371/journal.pgph.0000802

**Published:** 2023-10-26

**Authors:** Kristen Heitman, Sara E. Thomas, Owen Kelly, Stephanie M. Fanelli, Jessica L. Krok-Schoen, Menghua Luo, Christopher A. Taylor

**Affiliations:** 1 Medical Dietetics, The Ohio State University, Columbus, Ohio, United States of America; 2 Nutrition Science & Innovation, Abbott Nutrition, Columbus, Ohio, United States of America; 3 College of Osteopathic Medicine, Sam Houston State University, Huntsville, Texas, United States of America; 4 Health Sciences, The Ohio State University, Columbus, Ohio, United States of America; 5 Regulatory Affairs, Abbott Nutrition, Columbus, Ohio, United States of America; 6 Family Medicine, The Ohio State University, Columbus, Ohio, United States of America; University of Otago - Dunedin Campus: University of Otago, NEW ZEALAND

## Abstract

Little is known about the snacking patterns among adults with type 2 diabetes. The contribution of snacks to energy and nutrient intakes is important to further understand dietary patterns and glycemic control. The purpose of this study is to evaluate snack consumption among adults according to diabetes status in the United States. One NHANES 24-hour dietary recall for each participant collected between 2005–2016 was utilized for analysis (n = 23,708). Analysis of covariance was used to compare differences in nutrient and food groups intakes from snacks across levels of glycemic control, while controlling for age, race/ethnicity, income, marital status, and gender. Results of this analysis inform that adults with type 2 diabetes consume less energy, carbohydrates, and total sugars from snacks than adults without diabetes. Those with controlled type 2 diabetes consumed more vegetables and less fruit juice than other groups, yet adults with type 2 diabetes in general consumed more cured and luncheon meats than adults without diabetes or with prediabetes. Protein from all snacks for those without diabetes is higher than all other groups. This study elucidates common snacking patterns among US adults with diabetes and highlights the need for clinicians and policymakers to take snacking into consideration when evaluating and providing dietary recommendations.

## 1. Introduction

Lifestyle behaviors play a large role in the development and management of type 2 diabetes [1] and this is especially true for diet. Recent Dietary Guidelines for Americans focus on the overall dietary pattern since specific patterns (e.g. Mediterranean) have been associated with the prevention of chronic disease including type 2 diabetes [2]; thus, dietary quality may influence disease risk. Further complicating the field of nutrition research in diabetes is the range of studies; from intakes of specific foods or nutrients to overall dietary patterns. While there are advantages and disadvantages to both, they are equally required as nutritional guidelines for diabetes include recommendations for food groups/dietary patterns (e.g. Mediterranean, vegetarian) and specific nutrients (e.g. sodium, replacing saturated fat with unsaturated fats) [3]. Therefore, while dietary patterns provide a more holistic picture of dietary intake [4], information for each eating occasion and food decision is masked and may not provide insights into dietary modification/intervention for each eating occasion (meals and snacks).

People unknowingly make two hundred food-related decisions every day [5]. There are 5–6 eating occasions (3 main meals + 2–3 snacks) per day on average [6] suggesting that many of these food decisions may be related to snacks. Studies comparing meal frequency in people with type 2 diabetes are conflicting. A 16-year follow-up study of healthy men found that those who ate 1–2 times per day had a higher risk of type 2 diabetes than those who ate 3 times per day [7]. When factoring in body mass index, however, this association was not significant. When controlling for energy and macronutrient content, Kahleova et al. showed that just two larger meals each day were more effective for weight loss and improving metabolic parameters than 3 smaller meals with 3 snacks in people with type 2 diabetes [8, 9]. Under the setting of a clinical trial where the snack composition is well controlled like the previous study, these data may hold; however, after the trial participants return to their daily lives, they are likely to return to their previous nutritional behaviors. This may be an area that requires more real-world evidence.

Dietary Reference Intakes focus on the total daily intake and do not specify how much of a nutrient should be consumed during each eating occasion. In an earlier American Diabetes Association (ADA) position statement [10], the total carbohydrate intake per eating occasion, including snacks, was seen to be more important than the source and type. However, in the latest ADA Consensus report on Nutrition Therapy for Adults with Diabetes or Prediabetes, [3] snacks are not mentioned. Eating occasions are addressed through discussions of meal timing (especially for insulin users) and carbohydrate quality per eating occasion as principal considerations.

Snacking has been shown to contribute considerably to total intakes and is related to overall diet quality. Work by Nicklas et al. [11] shows that certain snacking habits (food choices) can affect overall dietary quality which indicates snacks significantly contribute to the overall dietary pattern and nutrient intake. This is supported by other work showing many snacks seem to be high in calories, yet poor in nutrients [12]. Therefore, snacks may negatively impact overall dietary patterns and nutrient intakes. While there is no standard definition for snacks [13], which can limit the interpretation of reported data, Kant & Graugard have observed a decrease in daily calories from the three main meals and a rise in calories from snacks over the last 40 years [6].

Every five years, the United States Department of Agriculture (USDA) releases an updated set of Dietary Guidelines for Americans recommending what foods should be included or excluded as part of a healthy dietary eating pattern [14, 15]. While these recommendations speak to the types and amounts of foods Americans should consume and also suggest choosing more nutrient-dense snacks, no national, expert energy or nutrient recommendations for each eating occasion, especially snacks, exist, even though snacks contribute significantly to the daily energy intake in the United States (US) [16]. Given the rising prevalence of diabetes in the US, and the role that dietary behaviors play in disease prevention and management, identifying those dietary behaviors that support health is an essential step in helping manage diabetes, and possibly delaying the progression from prediabetes to diabetes. Therefore, the aim of this study was to evaluate snack consumption, composition, and contribution to daily calories and nutrition, among adults without diabetes, with prediabetes, and type 2 diabetes in the US using the 2005–2016 National Health and Nutrition Examination Survey (NHANES).

## 2. Methods

### 2.1 Sample population

Data from 23,708 adults, over 30 years of age, from the 2005–2016 NHANES were analyzed to determine food and nutrient intake categories and their contribution of nutrients from snacking occasions in US adults stratified by diabetes status. NHANES is a federally funded surveillance system that monitors the health and nutrition status of the US population [17]. For survey data collected for this analysis, NHANES utilized a multistage, stratified sampling design to select participants from the noninstitutionalized population. Data were collected through questionnaires as well as physical and laboratory examinations performed in the mobile examination center. Subpopulations that are difficult to reach are oversampled and weighted to create a nationally representative data set. The survey collects demographic, socioeconomic, dietary, and health data via a mobile clinic. Demographic data, such as age, gender, marital status, and family income in relation to the federal poverty level, were collected during in-home interviews.

### 2.2 Ethical considerations

Public use data files from the National Health and Nutrition Examination Survey were obtained from the Centers for Disease Control and Prevention National Center for Health Statistics website for analysis. Participants gave written informed consent, and the National Center for Health Statistics’ Research Ethics Review Board approved all data collection methods.

### 2.3 Laboratory data: Classification levels of glycemic control

Glycated hemoglobin (GHb) was used to categorize diabetes status. During the visit to the NHANES mobile examination center, a blood sample was collected from consenting participants. Participants were categorized into levels of glycemic control according to their GHb percentage value: nondiabetes (< 5.7%, 39 mmol/mol); prediabetes (5.7–6.4%, 39-46mmol/mol); controlled diabetes (6.5–6.9%, 48–52 mmol/mol); and poorly controlled diabetes (≥ 7%, 53mmol/mol) [18]. Participants without data were excluded from the analyses (n = 2,314, 9%).

### 2.4 Dietary data

One recall per participant was assessed from 24-hour dietary recalls conducted by trained interviewers in the mobile examination center. Recalls utilized the Automated Multiple Pass Method, a validated, multistep interviewing technique shown to improve the accuracy of dietary intake data [19]. Participants reported the foods and beverages consumed from midnight to midnight from the prior day. Eating occasions were self-designated meals during the recalls and were reclassified as breakfast, lunch, dinner, or snack/other eating occasions for analysis. Individual nutrient and MyPlate equivalent intake estimates were generated using the Food and Nutrient Database for Dietary Studies [20] and the Food Patterns Equivalents Database [21]. To identify the types of foods contributing to snacking intakes, individual foods reported in the recall were classified according to the What We Eat in America food categories [22]. Participants who did not complete the dietary intake recall interview were excluded from analysis (n = 2,953, 11%).

### 2.5 Estimating snacking intakes

Total intakes of nutrients and MyPlate equivalents (eq) from snacking occasions were aggregated across all foods and beverages consumed from eating occasions described as snacks or other eating occasions. The proportion of the day’s intakes obtained from snacking occasions was computed as the sum of the nutrient or food group from snacks divided by the intake of the respective nutrient or food group from the total day. Average snack intakes were computed from total intakes from snacking occasions per the number of unique snack occasions reported. The highest energy snacking occasion was identified to represent the unique snacking occasion with the highest energy content per individual.

### 2.6 Statistical analysis

Complete dietary, demographic, and laboratory data were downloaded and imported into SPSS Complex Samples (version 24, IBM SPSS Inc., Chicago, IL). Multiple linear regressions were performed to examine intakes of snacks in relation to glycemic level among NHANES participants. Means and standard errors were generated for individual nutrient intakes and proportions of total intake for all reported snacking occasions by glycemic level. Analysis was controlled for age, race/ethnicity, income, marital status, and gender. Descriptive statistics were used to produce estimates of nutrients and food groups for an average snacking occasion, as well as for the average highest energy snacking occasion. The proportional contribution of snacking energy from USDA food categories was obtained as the snacking energy obtained from a food category divided by the total snacking energy reported, across levels of glycemic control. Sampling weights provided by the Centers for Disease Control were used to generate a nationally representative sample of population-based estimates. SPSS Complex Samples produced sample-based standard errors.

## 3. Results

### 3.1 Demographic characteristics according to diabetes status

Complete dietary intake data from 23,708 adults were evaluated from 2005–2016 NHANES. In general, males and females were equally distributed across each diabetes status. Compared to those without diabetes, those with prediabetes and type 2 diabetes were older (mean age of 50.5 years compared with ≥58.6 years, respectively, p<0.001). A larger percent of those without diabetes had a college degree (34.3%) compared to those with prediabetes (23.1%), controlled type 2 diabetes (19.1%), or poorly controlled diabetes (16.5%, p<0.001). Among the population surveyed, non-Hispanic whites represented the largest number in each diabetes status category followed by non-Hispanic blacks and Mexican Americans (p<0.001).

### 3.2 Nutrient Intakes from snacking occasions

Estimates of intakes from snacking occasions and the proportion of the day’s snack intakes are presented in Tables 1 and 2, respectively. Those without diabetes and with prediabetes, controlled type 2 diabetes, and poorly controlled type 2 diabetes consumed an average of 2 snacks per day, with no significant differences by diabetes status (p = 0.264). On the day of intake, combined snacking occasions contributed approximately 498 kcals of energy to the day’s intakes, which accounted for 22.4% of the total daily energy intake for adults without diabetes. Adults with prediabetes and diabetes (controlled or poorly controlled) consumed significantly less, accounting for 21.3%, 19.5%, and 19.6% (467, 435, and 423 kcal, p<0.001) of the day’s energy from snacks, respectively. For both groups with type 2 diabetes (controlled and poorly controlled), intakes of carbohydrates from snacks as well as the percent of the day’s carbohydrate intakes were significantly lower (59.0 g/22.6% and 57.8 g/23.1%, respectively, p = 0.002) compared to the nondiabetes group (67.1 g/25.3%). Added sugar intakes from snacking occasions followed similar trends as total carbohydrates, with the lowest intakes and contribution to the total day in those with type 2 controlled diabetes (35g/30.4%), and poorly controlled diabetes (33.8 g/31.0%) groups (p = 0.001). There were no differences in the amount of total fat and saturated fat from snacking occasions, but those in the nondiabetes group had significantly more protein than the prediabetes group (p = 0.023). The amount of protein from combined snacks was similar between the nondiabetes group and those with poorly controlled type 2 diabetes, but as a percentage of total daily protein intake, the poorly controlled type 2 diabetes group consumed significantly less protein (11.8% vs 13.0%, p = 0.004).

**Table 1:** Energy and nutrient intakes from all snacks and the percent of the day’s intakes of energy and nutrients obtained from snacks in people aged 30+ years with different diabetes status.

Mean Intakes	Diabetes status				p value^2^
	Nondiabetes	Prediabetes	Controlled diabetes	Poorly controlled diabetes	
Energy (kcal)	498 (7)^†^^‡^^§^	467 (10)*^§^	435 (21)*	423 (16)*^†^	<0.001
Protein (gm)	10.5 (0.2)^†^	9.6 (0.3)*	9.9 (0.7)	10.2 (0.5)	0.023
Percent of snack kcals	8.4%	8.2%	9.1%	9.6%	
Carbohydrate (gm)	67.1 (1.1)^§^^‡^	65.2 (1.5)^§^	59.0 (3.0)*	57.8 (2.4)*^†^	0.002
Percent of snack kcals	53.9%	55.8%	54.3%	54.7%	
Dietary fiber (gm)	3.3 (0.1)	3.2 (0.1)	3.1 (0.2)	3.2 (0.1)	0.461
Total fat (gm)	16.2 (0.3)	15.8 (0.4)	15.6 (1.0)	15.1 (0.8)	0.450
Percent of snack kcals	29.3%	30.4%	32.3%	32.1%	
Total saturated fat (gm)	5.4 (0.1)	5.4 (0.2)	5.2 (0.4)	5.1 (0.3)	0.717
Percent of snack kcals	9.8%	10.4%	10.8%	10.9%	
Vitamin E, ATE (mg)	1.9 (0.1)^§^	1.8 (0.1)	1.8 (0.1)	1.6 (0.1)*	0.003
Vitamin A, RAE (ug)	95.0 (3.6)	100.2 (9.2)	124.2 (20.4)	90.3 (6.4)	0.288
Vitamin B6 (mg)	0.36 (0.01)^†^^‡^^§^	0.32 (0.01)*^‡^	0.27 (0.02)*^†^	0.3 (0.02)*	<0.001
Folate, DFE (ug)	82.4 (1.8)^‡^	77.6 (2.4)^‡^	66.8 (4.2)*^†^	78 (4.5)	0.003
Vitamin B12 (ug)	0.73 (0.03)	0.66 (0.03)	0.64 (0.06)	0.68 (0.04)	0.186
Vitamin C (mg)	21.1 (0.7)^†^	17.8 (0.9)*	17.1 (1.8)	18.5 (1.6)	0.004
Vitamin D (ug)	0.6 (0.02)	0.54 (0.03)	0.69 (0.13)	0.66 (0.07)	0.183
Calcium (mg)	230 (4)^†^	212 (5)*	233 (15)	233 (9)	0.015
Phosphorus (mg)	245 (4)^†^	220 (6)*	233 (14)	233 (10)	<0.001
Magnesium (mg)	82.1 (1.2)^†^^‡^^§^	74.1 (1.8)*	70.7 (3.3)*	75.8 (2.5)*	<0.001
Iron (mg)	2.3 (0.01)	2.2 (0.1)	2.1 (0.1)	2.2 (0.1)	0.328
Zinc (mg)	1.7 (0.01)^†^	1.6 (0.01)*	1.6 (0.1)	1.6 (0.1)	0.018
Copper (mg)	0.35 (0.01)^†^	0.32 (0.01)*	0.32 (0.02)	0.34 (0.01)	0.023
Sodium (mg)	436 (8)	432 (13)	457 (29)	455 (22)	0.720
Potassium (mg)	565 (10)^†^^‡^^§^	523 (14)*	496 (25)*	523 (22)*	0.004
Selenium (ug)	12.1 (0.3)	11.2 (0.3)	10.9 (0.9)	11.3 (0.6)	0.117
Alcohol (gm)	7.8 (0.3)^†^^‡^	5.1 (0.4)*	4.1 (0.7)*	3.8 (0.5)	<0.001
Total fruit (c)	0.41 (0.01)^‡^	0.37 (0.02)	0.32 (0.03)*	0.41 (0.03)	0.003
Fruit juices (c)	0.09 (0.01)^†^^‡^	0.07 (0.01)*^‡^	0.03 (0.01)*^†^	0.07 (0.01)	<0.001
Whole fruits (c)	0.32 (0.01)	0.30 (0.02)	0.29 (0.03)	0.34 (0.03)	0.498
Total vegetables (c)	0.11 (0.01)^‡^	0.12 (0.01)	0.18 (0.03)*	0.09 (0.01)	0.005
Starchy vegetables (c)	0.05 (0.01)	0.05 (0.01)	0.07 (0.01)	0.04 (0.01)	0.229
Total grains (oz)	1.0 (0.02)	1.0 (0.03)	1.0 (0.08)	1.0 (0.05)	0.972
Whole grains (oz)	0.2 (0.01)	0.2 (0.01)	0.2 (0.03)	0.2 (0.02)	0.922
Refined grains (oz)	0.8 (0.02)	0.8 (0.03)	0.8 (0.08)	0.8 (0.05)	0.903
Total protein foods (oz)	0.76 (0.03)	0.68 (0.04)	0.62 (0.07)	0.72 (0.06)	0.083
Meat products (oz)	0.22 (0.01)	0.21 (0.01)	0.20 (0.03)	0.24 (0.03)	0.594
Total milk and dairy (c)	0.33 (0.01)^†^	0.29 (0.01)*	0.36 (0.05)	0.33 (0.03)	0.041
Fluid milk (c)	0.21 (0.01)^†^	0.18 (0.01)*	0.22 (0.04)	0.22 (0.03)	0.045
Added sugars (g)	27.2 (0.6)^§^	27.8 (0.9)^§^	23.2 (1.8)	21.4 (1.5)*^†^	0.001
Percent of snack kcals	21.8%	23.8%	21.3%	20.2%	

*Pairwise comparison was statistically significant (*P<* 0.05) compared with the Nondiabetes group

^†^Pairwise comparison was statistically significant (*P<* 0.05) compared with the Prediabetes group

^‡^Pairwise comparison was statistically significant (*P<* 0.05) compared with the Controlled diabetes group

^§^Pairwise comparison was statistically significant (*P<* 0.05) compared with the Poorly controlled diabetes group

GHb classifications: nondiabetes (GHb: <5.7% (<39 mmol/mol)); prediabetes (GHb: 5.7–6.4% (39–46 mmol/mol)); controlled diabetes (GHb: 6.5–6.9% (48–52 mmol/mol)); and poorly controlled diabetes (GHb: ≥7% (≥53 mmol/mol)). Multiple linear regression was used to generate mean (SE) controlled for age, race/ethnicity, income, marital status and gender.

Abbreviations: ATE: alpha tocopherol equivalents, RAE: retinol activity equivalent; DFE: dietary folate equivalents.

**Table 2:** Proportion of the day’s total intakes of energy and nutrients obtained from snack occasions in people aged 30+ with different diabetes status.

Mean Intakes	Diabetes status * ^†^ ^‡^ ^§^				p value^2^
	Nondiabetes	Prediabetes	Controlled diabetes	Poorly controlled diabetes	
Energy (kcal)	22.4 (0.3)^†^^‡^^§^	21.3 (0.4)*	19.5 (0.7)*	19.6 (0.6)*	<0.001
Protein (gm)	13.0 (0.2)^§^	12.2 (0.3)	11.7 (0.6)	11.8 (0.4)*	0.004
Carbohydrate (gm)	25.3 (0.3)^‡^^§^	24.4 (0.4)	22.6 (0.9)*	23.1 (0.7)*	0.003
Dietary fiber (gm)	18.7 (0.3)	18.3 (0.4)	17.6 (0.8)	18.4 (0.6)	0.539
Total fat (gm)	19.1 (0.3)^§^	18.5 (0.5)	17.4 (0.8)	16.8 (0.7)*	0.004
Total saturated fat (gm)	19.8 (0.3)^‡^^§^	19.1 (0.5)	17.6 (0.8)*	17.5 (0.8)*	0.006
Vitamin E, ATE (mg)	19.5 (0.3)	18.9 (0.4)	18.9 (0.9)	18.1 (0.7)	0.218
Vitamin A, RAE (ug)	15.4 (0.3)	14.6 (0.4)	15.3 (1.1)	14.4 (0.7)	0.304
Vitamin B6 (mg)	15.7 (0.2)^†^^‡^^§^	14.5 (0.4)*	13.2 (0.7)*	14.1 (0.5)*	<0.001
Folate, DFE (ug)	16.3 (0.2)^‡^^§^	15.5 (0.4)	13.7 (0.7)*	14.7 (0.6)*	<0.001
Vitamin B12 (ug)	13.6 (0.3)	12.6 (0.4)	12.4 (0.9)	13.1 (0.7)	0.134
Vitamin C (mg)	19.5 (0.4)	18.8 (0.6)	20.1 (1.5)	19.7 (1.1)	0.746
Vitamin D (ug)	14.1 (0.4)	13.5 (0.5)	13.0 (1.5)	14.7 (0.8)	0.499
Calcium (mg)	24.5 (0.3)	23.7 (0.4)	23.3 (0.9)	24.2 (0.7)	0.222
Phosphorus (mg)	17.4 (0.2)^†^	16.2 (0.4)*	15.8 (0.7)	16.1 (0.6)	0.002
Magnesium (mg)	25.0 (0.3)^‡^	24.1 (0.4)	22.9 (0.8)*	24.2 (0.5)	0.010
Iron (mg)	15.6 (0.2)	15.1 (0.4)	14.2 (0.7)	14.9 (0.6)	0.100
Zinc (mg)	15.5 (0.2)^†^	14.4 (0.4)*	14.1 (0.7)	14.3 (0.5)	0.012
Copper (mg)	25.3 (0.3)	24.3 (0.4)	23.9 (0.9)	24.7 (0.5)	0.064
Sodium (mg)	13.2 (0.2)	12.7 (0.3)	12.0 (0.6)	12.5 (0.5)	0.087
Potassium (mg)	19.9 (0.3)	19.3 (0.4)	18.1 (0.8)	19.0 (0.6)	0.054
Selenium (ug)	10.8 (0.2)^§^	10.2 (0.3)	9.5 (0.6)	9.6 (0.4)*	0.004
Alcohol (gm)	52.0 (1.1)	51.7 (2.8)	51.8 (6.5)	49.4 (5)	0.968
Total fruit (c)	35.5 (0.7)^§^	35.4 (1)	3.03 (2.1)	41.9 (2)*	0.005
Fruit juices (c)	26.5 (0.9)^†^	22.1 (1.1)*	21.1 (2.8)	26.5 (2.4)	0.011
Whole fruits (c)	39.0 (0.8)^§^	40.3 (1.3)	37.8 (2.7)	47.0 (2.1)*	0.001
Total vegetables (c)	6.8 (0.2)^§^	7.2 (0.4)	8.5 (1.1)	5.2 (0.4)*	<0.001
Starchy vegetables (c)	11.7 (0.5)	12.1 (0.8)	14.5 (2.4)	10.4 (1.1)	0.332
Total grains (oz)	15.1 (0.3)	14.7 (0.4)	13.9 (0.9)	14.1 (0.7)	0.245
Whole grains (oz)	21.2 (0.6)	20.3 (1)	19.6 (2.5)	22.5 (1.7)	0.523
Refined grains (oz)	15.1 (0.3)	14.9 (0.5)	13.7 (0.9)	13.9 (0.7)	0.189
Total protein foods (oz)	9.9 (0.3)^†^^‡^^§^	8.8 (0.5)*	8.0 (0.7)*	8.4 (0.6)*	0.003
Meat products (oz)	4.2 (0.2)	3.6 (0.3)	3.7 (0.6)	3.8 (0.3)	0.164
Total milk and dairy (c)	21.8 (0.4)	20.9 (0.6)	21.9 (1.6)	21.6 (1.1)	0.714
Fluid milk (c)	27.1 (0.5)	27.0 (0.8)	24.9 (1.7)	27.0 (1.4)	0.607
Added sugars (g)	34.0 (0.4)^§^	34.1 (0.7)	30.9 (1.7)	31.0 (1.1)*	0.019

*Pairwise comparison was statistically significant (*P<* 0.05) compared with the Nondiabetes group

^†^Pairwise comparison was statistically significant (*P<* 0.05) compared with the Prediabetes group

^‡^Pairwise comparison was statistically significant (*P<* 0.05) compared with the Controlled diabetes group

^§^Pairwise comparison was statistically significant (*P<* 0.05) compared with the Poorly controlled diabetes group

GHb classifications: nondiabetes (GHb: <5.7% (<39 mmol/mol)); prediabetes (GHb: 5.7–6.4% (39–46 mmol/mol)); controlled diabetes (GHb: 6.5–6.9% (48–52 mmol/mol)); and poorly controlled diabetes (GHb: ≥7% (≥53 mmol/mol)). Multiple linear regression was used to generate mean (SE), controlled for age, race/ethnicity, income, marital status and gender.

Abbreviations: ATE: alpha tocopherol equivalents, RAE: retinol activity equivalent; DFE: dietary folate equivalents.

Intakes of vitamin B_6_, and magnesium from snacks, were significantly lower in those in the controlled and poorly controlled type 2 diabetes groups compared to those in the nondiabetes group (p<0.001). However, as a percentage of total daily magnesium intake, there was only a significant difference between the nondiabetes and controlled type 2 diabetes group, whereas significance remained for vitamin B_6_ (p<0.001).

### 3.3 Food group and nutrient profile of an average snack

On the day of intake, adults with poorly controlled type 2 diabetes consumed significantly less energy (p = 0.002), carbohydrate (p<0.002), and added sugars (p<0.001) per average snacking occasion compared to adults with prediabetes or without diabetes (Table 3). For the average snack, adults with controlled type 2 diabetes consumed modestly but less total protein foods (0.29 oz. eq.) compared to those without diabetes (0.36 oz. eq., p = 0.14), yet significantly higher amounts of total vegetables (including legumes) (0.1 cup eq.) than those without diabetes (0.05 cup eq.), prediabetes (0.07 cup eq.) or poorly controlled type 2 diabetes (0.05 cup eq., p = 0.004). People without diabetes and with prediabetes consumed significantly more added sugars compared to those with poorly controlled type 2 diabetes (p<0.001). Regardless of diabetes status, refined grains accounted for >80% of all grains when examining the average and highest snack. The proportion of whole grains was not significantly different by diabetes status. Additionally, adults with controlled type 2 diabetes consumed significantly less fruit juice than any other group per average snacking occasion (p<0.001). In all groups, the average snack did not contain dark green vegetables, starchy vegetables (excluding white potatoes), legumes, organ meat or seafood high in omega-3 fatty acids.

**Table 3:** Energy and nutrient content of the average snack occasions in people aged 30+ with different diabetes status.

Mean Intakes	Diabetes status				p value^2^
	Nondiabetes	Prediabetes	Controlled diabetes	Poorly controlled diabetes	
Energy (kcal)	244 (2.4)^§^	243 (4.8)^§^	240 (14.2)	217 (6.7)*^†^	0.002
Protein (gm)	5.1 (0.07)	5.0 (0.14)	5.4 (0.44)	5.2 (0.21)	0.817
Carbohydrate (gm)	32.6 (0.37)^§^	33.7 (0.72)^§^	32.4 (1.84)	29.7 (0.83)*^†^	0.002
Dietary fiber (gm)	1.6 (0.03)	1.7 (0.06)	1.7 (0.1)	1.7 (0.07)	0.461
Total fat (gm)	7.9 (0.12)	8.3 (0.24)	8.9 (0.72)	7.8 (0.37)	0.285
Total saturated fat (gm)	2.6 (0.05)	2.8 (0.09)	3 (0.31)	2.6 (0.15)	0.161
Vitamin E, ATE (mg)	0.95 (0.03)^§^	0.93 (0.04)	1.04 (0.08)^§^	0.82 (0.04)*^‡^	0.023
Vitamin A, RAE (ug)	45.5 (1.8)	51.0 (5.3)	78.4 (20.4)	46.0 (3.4)	0.207
Vitamin B6 (mg)	0.18 (0.01)^‡^	0.17 (0.01)	0.14 (0.01)*	0.16 (0.01)	0.006
Folate, DFE (ug)	40.7 (0.8)	41.4 (1.3)	38.2 (2.5)	40.9 (2.4)	0.648
Vitamin B12 (ug)	0.35 (0.01)	0.34 (0.02)	0.38 (0.06)	0.35 (0.02)	0.958
Vitamin C (mg)	10.2 (0.3)	8.9 (0.5)	9.2 (1.0)	9.5 (0.8)	0.125
Vitamin D (ug)	0.35 (0.01)	0.31 (0.01)	0.51 (0.15)	0.39 (0.04)	0.049
Calcium (mg)	88.5 (1.4)	84.4 (2.2)	112.5 (13.5)	92.8 (4.2)	0.109
Phosphorus (mg)	118 (1)	113 (3)	131 (11)	119 (5)	0.311
Magnesium (mg)	34.4 (0.4)	32.7 (0.8)	33 (1.7)	32 (1.1)	0.057
Iron (mg)	1.1 (0.02)	1.2 (0.04)	1.2 (0.08)	1.2 (0.05)	0.446
Zinc (mg)	0.81 (0.01)	0.79 (0.02)	0.85 (0.06)	0.81 (0.04)	0.691
Copper (mg)	0.14 (0.002)	0.13 (0.003)	0.14 (0.008)	0.13 (0.005)	0.277
Sodium (mg)	201 (3)	213 (6)	237 (16)	220 (10)	0.023
Potassium (mg)	270 (3)	264 (6)	275 (18)	264 (8)	0.782
Selenium (ug)	6.0 (0.1)	5.9 (0.2)	6.0 (0.5)	6.1 (0.3)	0.959
Alcohol (gm)	3.9 (0.2)^†^^‡^^§^	2.8 (0.2)*^§^	2.0 (0.4)*	1.8 (0.2)^†^^§^	<0.001
Total fruit (c)	0.2 (0.01)	0.18 (0.01)	0.17 (0.02)^§^	0.22 (0.02)^‡^	0.025
Fruit juices (c)	0.05 (0.01)^†^^‡^	0.04 (0.01)*^‡^	0.02 (0.01)*^†^^§^	0.04 (0.01)^‡^	<0.001
Whole fruits (c)	0.15 (0.01)	0.15 (0.01)	0.15 (0.02)	0.18 (0.01)	0.272
Total vegetables (c)	0.05 (0.01)^‡^	0.07 (0.01)^§^	0.10 (0.02)^§^	0.05 (0.01)^†^^‡^	0.004
Starchy vegetables (c)	0.03 (0.01)	0.03 (0.01)	0.03 (0.01)	0.02 (0.01)	0.266
Total grains (oz)	0.48 (0.01)	0.52 (0.02)	0.55 (0.04)	0.53 (0.03)	0.028
Whole grains (oz)	0.08 (0.01)	0.09 (0.01)	0.08 (0.01)	0.09 (0.01)	0.713
Refined grains (oz)	0.40 (0.01)	0.43 (0.02)	0.47 (0.04)	0.44 (0.02)	0.060
Total protein foods (oz)	0.37 (0.01)	0.36 (0.02)	0.30 (0.03)	0.35 (0.03)	0.140
Meat products (oz)	0.11 (0.01)	0.11 (0.01)	0.1 (0.01)	0.12 (0.01)	0.466
Total milk and dairy (c)	0.15 (0.01)	0.14 (0.01)	0.23 (0.05)	0.17 (0.01)	0.220
Fluid milk (c)	0.1 (0.01)	0.09 (0.01)	0.14 (0.04)	0.11 (0.01)	0.361
Added sugars (g)	13.0 (0.3)^§^	14.2 (0.5)^§^	12.2 (0.9)	10.5 (0.6)*^†^	<0.001

*Pairwise comparison was statistically significant (*P<* 0.05) compared with the Nondiabetes group

^†^Pairwise comparison was statistically significant (*P<* 0.05) compared with the Prediabetes group

^‡^Pairwise comparison was statistically significant (*P<* 0.05) compared with the Controlled diabetes group

^§^Pairwise comparison was statistically significant (*P<* 0.05) compared with the Poorly controlled diabetes group

Multiple linear regression was used to generate mean (SE), controlled for age, race/ethnicity, income, marital status and gender.

Abbreviations: ATE: alpha tocopherol equivalents, RAE: retinol activity equivalent; DFE: dietary folate equivalents.

### 3.4 Food group and nutrient profile of highest energy snack

The highest calorie snack for those with poorly controlled type 2 diabetes had significantly less energy (p = 0.003) and carbohydrates (p = 0.027), compared to the highest calorie snack for adults with prediabetes or without diabetes (Table 4). Adults with poorly controlled type 2 diabetes consumed significantly fewer total vegetables (p = 0.002) compared to those with prediabetes and controlled type 2 diabetes. Adults with controlled type 2 diabetes consumed significantly less fruit juice during the highest energy snack compared to people without diabetes or prediabetes (p = 0.001). Like the average snacking occasion, people with poorly controlled type 2 diabetes consumed significantly less added sugar during the highest calorie snack compared to those with prediabetes and those without diabetes (p<0.010).

**Table 4:** Energy and nutrient content of the highest calorie snack in people aged 30+ with different diabetes status.

Mean Intakes	Diabetes status				p value^2^
	Nondiabetes	Prediabetes	Controlled diabetes	Poorly controlled diabetes	
Energy (kcal)	373 (5)^§^	362 (7)^§^	354 (18)	330 (12)*^†^	0.003
Protein (gm)	8.0 (0.2)	7.7 (0.2)	8.1 (0.6)	8.0 (0.4)	0.589
Carbohydrate (gm)	46.9 (0.7)^§^	47.8 (1.2)^§^	45.1 (2.3)	42.7 (1.6)*^†^	0.027
Dietary fiber (gm)	2.3 (0.01)	2.4 (0.1)	2.3 (0.1)	2.4 (0.1)	0.627
Total fat (gm)	12.9 (0.2)	13.1 (0.3)	13.6 (1.0)	12.4 (0.6)	0.675
Total saturated fat (gm)	4.2 (0.1)	4.5 (0.1)	4.6 (0.4)	4.1 (0.2)	0.281
Vitamin E, ATE (mg)	1.4 (0.01)	1.4 (0.1)	1.5 (0.1)	1.3 (0.1)	0.284
Vitamin A, RAE (ug)	65.4 (3.5)	75.8 (10.3)	102.8 (21.4)	67.1 (5.7)	0.339
Vitamin B6 (mg)	0.24 (0.01)^‡^^§^	0.23 (0.01)^‡^	0.2 (0.01)*^†^	0.21 (0.01)*	0.001
Folate, DFE (ug)	59.8 (1.3)	60.6 (3.1)	53.5 (3)	60.5 (4)	0.205
Vitamin B12 (ug)	0.5 (0.02)	0.49 (0.03)	0.51 (0.06)	0.51 (0.04)	0.987
Vitamin C (mg)	11.8 (0.5)	10.5 (0.7)	11.5 (1.8)	10.9 (1.0)	0.417
Vitamin D (ug)	0.53 (0.02)^†^	0.46 (0.02)*	0.69 (0.16)	0.61 (0.08)	0.024
Calcium (mg)	130 (3)	124 (4)	151 (16)	136 (9)	0.216
Phosphorus (mg)	181 (3)	170 (5)	186 (13)	177 (8)	0.170
Magnesium (mg)	49.6 (0.7)^†^	46.1 (1.4)*	45.1 (2.3)	45.6 (1.8)	0.007
Iron (mg)	1.7 (0.03)	1.8 (0.1)	1.7 (0.1)	1.8 (0.1)	0.662
Zinc (mg)	1.2 (0.01)	1.2 (0.01)	1.2 (0.1)	1.3 (0.1)	0.512
Copper (mg)	0.2 (0.01)	0.2 (0.01)	0.2 (0.01)	0.2 (0.01)	0.305
Sodium (mg)	310 (7)	324 (10)	363 (28)	344 (20)	0.080
Potassium (mg)	365 (6)	351 (10)	360 (20)	344 (12)	0.382
Selenium (ug)	9.7 (0.3)	9.2 (0.3)	9.3 (0.7)	9.5 (0.6)	0.569
Alcohol (gm)	6.5 (0.3)^†^^‡^^§^	4.3 (0.4)*	3.7 (0.7)*	3.2 (0.5)*	<0.001
Total fruit (c)	0.22 (0.01)	0.2 (0.01)	0.18 (0.02)	0.24 (0.02)	0.019
Fruit juices (c)	0.06 (0.01)^‡^	0.05 (0.01)^‡^	0.02 (0.01)*^†^	0.05 (0.01)	0.001
Whole fruits (c)	0.16 (0.01)	0.15 (0.01)	0.16 (0.02)	0.19 (0.02)	0.334
Total vegetables (c)	0.08 (0.01)	0.10 (0.01)^§^	0.12 (0.02)^§^	0.06 (0.01)^†^^‡^	0.002
Starchy vegetables (c)	0.04 (0.01)	0.04 (0.01)	0.05 (0.01)	0.03 (0.01)	0.130
Total grains (oz)	0.75 (0.02)	0.81 (0.03)	0.84 (0.08)	0.82 (0.05)	0.178
Whole grains (oz)	0.12 (0.01)	0.13 (0.02)	0.14 (0.03)	0.14 (0.02)	0.474
Refined grains (oz)	0.63 (0.02)	0.67 (0.03)	0.70 (0.08)	0.68 (0.04)	0.412
Total protein foods (oz)	0.63 (0.02)	0.57 (0.04)	0.52 (0.06)	0.63 (0.05)	0.093
Meat products (oz)	0.19 (0.01)	0.18 (0.01)	0.19 (0.03)	0.22 (0.03)	0.717
Total milk and dairy (c)	0.23 (0.01)	0.22 (0.01)	0.3 (0.06)	0.25 (0.02)	0.381
Fluid milk (c)	0.15 (0.01)	0.14 (0.01)	0.18 (0.04)	0.17 (0.02)	0.537
Added sugars (g)	18.7 (0.5)	20.1 (0.8)^§^	17.3 (1.3)	15.6 (1.3)^†^	0.010

*Pairwise comparison was statistically significant (*P<* 0.05) compared with the Nondiabetes group

^†^Pairwise comparison was statistically significant (*P<* 0.05) compared with the Prediabetes group

^‡^Pairwise comparison was statistically significant (*P<* 0.05) compared with the Controlled diabetes group

^§^Pairwise comparison was statistically significant (*P<* 0.05) compared with the Poorly controlled diabetes group

GHb classifications: nondiabetes (GHb: <5.7% (<39 mmol/mol)); prediabetes (GHb: 5.7–6.4% (39–46 mmol/mol)); controlled diabetes (GHb: 6.5–6.9% (48–52 mmol/mol)); and poorly controlled diabetes (GHb: ≥7% (≥53 mmol/mol)). Multiple linear regression was used to generate mean (SE), controlled for age, race/ethnicity, income, marital status and gender.

Abbreviations: ATE: alpha tocopherol equivalents, RAE: retinol activity equivalent; DFE: dietary folate equivalents.

### 3.5 Food sources of energy from snacks

**Fig 1:**
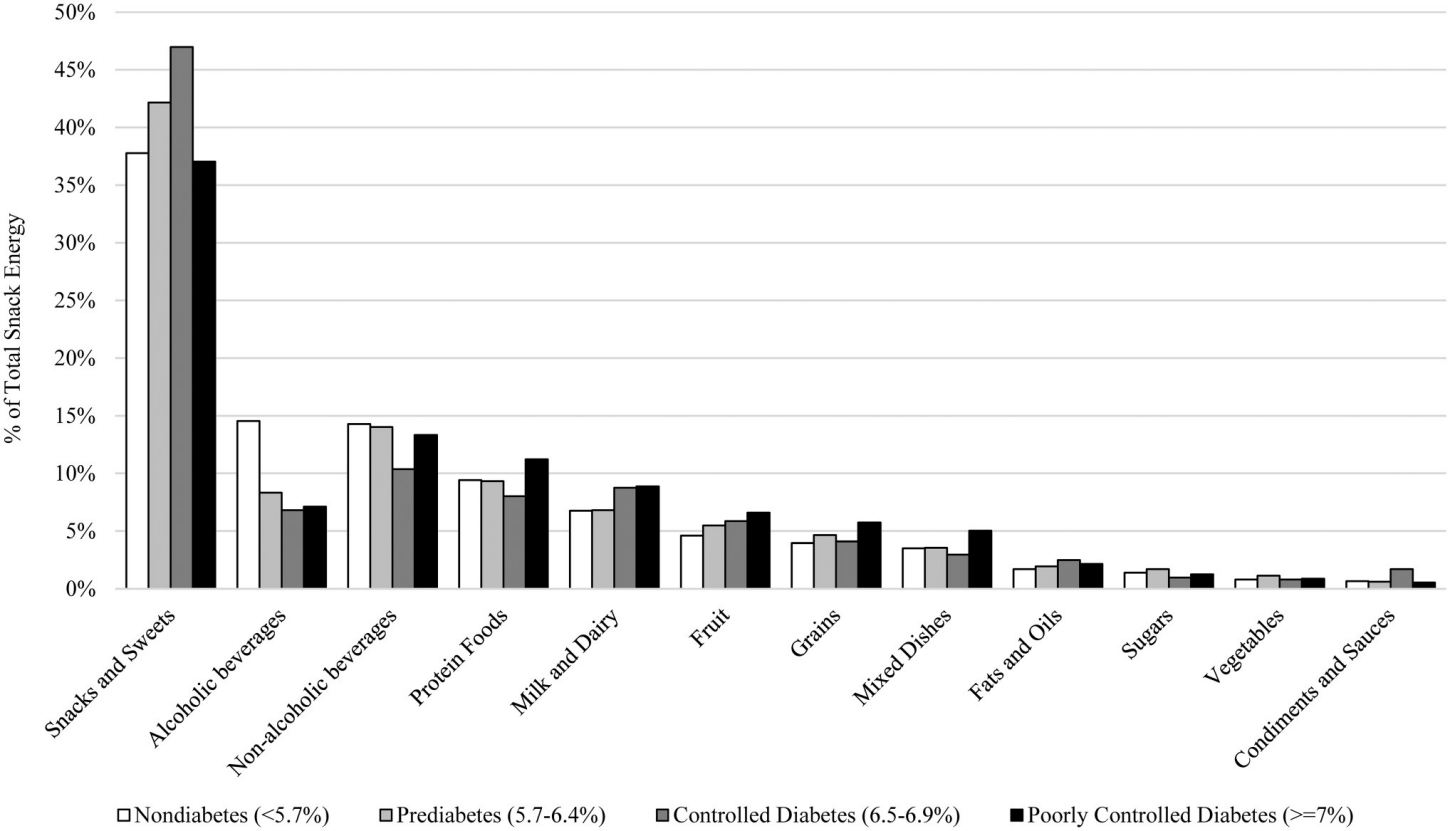
Proportion of food sources of energy consumed during snacking occasions across levels of glycemic control.

Across all adults, snacks and sweets and non-alcoholic beverages accounted for approximately half of the energy consumed during snacking occasions (Fig 1). Alcoholic beverages contributed to 14.6% of the energy from snacks in the nondiabetes group, which was two-fold greater than for the other groups. The lowest proportion of non-alcoholic beverages was noted in the controlled diabetes group, but they also obtained 47% of snack energy from the snacks and sweets category. Fruits and vegetables accounted for 5% of energy from snacking occasions.

## 4. Discussion

Lifestyle modification, such as a change in eating pattern, is an effective method for prevention and management of diabetes [23]. In fact, individuals with diabetes who have poor diet quality have nearly three-fold odds chance of poor glycemic control [22]. Post-prandial glucose response after each eating occasion, including snacks, may influence GHb [24]. Therefore, understanding snack quality as it fits into overall dietary patterns in individuals according to diabetes status is needed to elucidate snacking recommendations. The results of our study are in line with other studies [12, 16] and demonstrate that snacks account for a substantial amount (19.5–22.4%) of daily energy intake. Regardless of diabetes status, our study shows that snacks contribute very little nutritional quality to the overall diet and may result in poorer dietary patterns.

### 4.1 Snacking according to diabetes status

Few studies address the association between snacking and diabetes status. Mekary, et al. analyzed cohorts of men and women and determined snacks beyond the three main meals per day increased the risk for Type 2 diabetes [7, 25]. Other research concludes that snacking beyond three main meals per day has little impact on glycemic control, but other factors such as timing and overall energy intake do affect glycemic control [26, 27]. In a study with 163 participants who had insulin-treated diabetes, there were no correlations between the use of snacks and GHb [26]. However, as elevated postprandial glucose may contribute to glycated hemoglobin (GHb) [28], it seems logical that the glycemic response after each eating occasion should be addressed. In the present study, regardless of diabetes status, adults consumed an average of 2 snacks on the day of intake. Interestingly, adults without diabetes consumed significantly more calories in the form of snacks, and snacks made up a higher portion of energy intake compared to all other groups. While the number of snacks was similar between groups, the energy content of snacks in relation to the total daily energy consumed varied on the day of intake and may play a role in glycemic control. As such, the information from this study can contribute towards making specific snack-related nutrition recommendations.

Increased intake of refined carbohydrates and sugars, in addition to decreased fiber, parallel the increased prevalence of type 2 diabetes [29–32]. In our cohort, dietary fiber intake from snack foods was similarly low ranging from 1.6–1.7 g/day along with a poor intake of whole grains while total sugar intake was relatively high (17.1–20.3 g/day), suggesting poor overall carbohydrate quality regardless of diabetes status. The average snacks consumed were higher in carbohydrates, total sugar, and added sugar for those without diabetes and with prediabetes than those with type 2 diabetes. Although the contribution of added sugars is still relatively high, this suggests that adults with type 2 diabetes may be taking steps to limit carbohydrates and sugar in their diets as stressed in medical nutrition therapy guidelines. However, those with prediabetes consumed more lower quality carbohydrates on the day of intake indicating a potential gap and opportunity for increased nutrition education in this group. The 2019 ADA Consensus Report now includes recommendations for people with prediabetes whereas previous ADA nutrition position papers did not [3], emphasizing the increased importance in prevention (not just treatment) of type 2 diabetes. The consensus report outlines the importance of implementing intensive lifestyle/behavior interventions to improve eating habits and increasing exercise to induce weight loss which is associated with a reduction in the incidence of type 2 diabetes in adults with prediabetes. Given the poor quality of carbohydrates consumed in this study, especially in those with prediabetes, it would be advantageous to incorporate specific snacking guidelines.

Protein is an essential macronutrient than can support satiety, and aid in the maintenance of lean body mass [12, 33–36]. Despite the high amount of sugar and refined carbohydrates consumed during snacks in all groups, the higher protein intake in people without diabetes during snacks may aid in satiety and reduce overeating at other meals. People without diabetes also consumed less processed meats as well as more nuts, seeds, and soy, while those with type 2 diabetes consumed more cured and luncheon meats during snacks. In addition to protein quantity, the sources of protein and their contributions with other macronutrients may impact glycemic response. In fact, it’s been shown that consuming glucose along with lean protein (such as lean beef and turkey) results in a lower blood glucose response compared to consuming glucose alone in people with type 2 diabetes [37]. Although glucose is the primary stimulus for insulin release, protein and amino acids can enhance the release of insulin leading to faster clearance of glucose from the blood [38]. Higher protein intake (30% of total daily calories) in people with type 2 diabetes is associated with lower GHb [39]. Thus, people with type 2 diabetes may benefit from increasing the protein content of their snacks, focusing on lean protein choices and consuming less processed meats, while maintaining overall energy density of their snack.

The literature is lacking to define the snack consumption, composition and contribution to daily calories and nutrition over a 24-hour period stratified by levels of glycemic control in a US population cohort. Overall, adults with type 2 diabetes consumed less energy, carbohydrates, and total sugars from snacks than adults without diabetes. Those with controlled type 2 diabetes consumed more vegetables and less fruit juice than other groups, yet adults with diabetes in general consumed more cured and luncheon meats than adults without diabetes and with prediabetes. Finally, adults without diabetes consumed more protein from snacks, including less processed meats. Regardless of diabetes status, the nutrition content of snacks was poor including mostly refined grains, added sugar, and lacked dark green vegetables, legumes, or seafood high in omega-3 fatty acids.

### 4.2 Strengths/Limitations

This study was performed utilizing a large national dataset, which contributes to the validity and reproducibility of our findings. In the absence of consistent fasting blood glucose values, GHb was the objective measures used to determine diabetes status. While it is less influenced by fasting status, it may not be as sensitive of a measure for prediabetes.

While self-report dietary data has limitations regarding correct reporting of type and quantity of food consumed, self-defining snacking occasions may be a strength [13, 40]. Previous studies have defined snacking by time of day, caloric level, foods consumed outside of the standard three meals per day, or have chosen to exclude certain beverages, or beverages altogether [7, 14, 16, 25, 27]. While defining snacks in these ways is objective, it does not address snacking as a cultural construct, which allows for diverse interpretation based on cultural norms [13]. This analysis will not account for individuals who consume small, frequent meals and should be considered when interpreting the findings related to snacks. While there is potential for snacking intakes to change over this period of 2005–2016, the mean intakes of energy and macronutrients from snacks across the data cycles was generally consistent. Due to the cross-sectional nature of this data set, the scope of interpretation is limited to difference based on the foods and beverages reported on the day of intake. These intakes cannot be assumed to reflect usual intakes, including usual snacking patterns. This study contributes significantly to the literature on snacking and diabetes. Future work could explore these trends in relation to total energy intakes and obesity to evaluate the covariance with diabetes status.

## 5. Summary

Snacks make up ~25% of 24-hour energy intake among US adults, contributing a meal’s worth of energy to overall intakes without conforming to central tenets of the Dietary Guidelines or the ADA diabetes treatment guidelines. Furthermore, snack composition differs when stratified by GHb. This data emphasizes the need for clinicians to assess snack intake, both in quantity and quality. While recommendations on frequency must be individualized, nutrition interventions must target nutrient-dense snacking, including snacks higher in protein, fiber, vegetables, and lower in simple sugars, and refined carbohydrates, while maintaining caloric intake. Lastly, this research informs public health administrators and nutrition policy, supporting the need for snacking recommendations.
